# Supplementary material for: Prevalence and predictors of HIV and sexually transmitted infections among vulnerable women engaged in sex work: Findings from the Kyaterekera Project in Southern Uganda
Source: PLoS One. 2022 Sep 29;17(9):e0273238. doi: 10.1371/journal.pone.0273238 (PMC9522279; doi:10.1371/journal.pone.0273238)
Supplement: Attachment — Submitted filename: Response to Reviewers.docx [file pone.0273238.s002.docx]

**Prevalence and Predictors of HIV and Sexually Transmitted Infections among Vulnerable Women Engaged in Sex Work: Findings from the Kyaterekera Project in Southern Uganda**

**Response to Reviewers’ Comments**

**Academic Editor**

1. Please ensure that your manuscript meets PLOS ONE's style requirements, including those for file naming. The PLOS ONE style templates can be found at
   https://journals.plos.org/plosone/s/file?id=wjVg/PLOSOne_formatting_sample_main_body.pdf and 
   https://journals.plos.org/plosone/s/file?id=ba62/PLOSOne_formatting_sample_title_authors_affiliations.pdf

***Response: We have followed the PLOS ONE’s manuscript required style.***

2. Please include a complete copy of PLOS’ questionnaire on inclusivity in global research in your revised manuscript. Our policy for research in this area aims to improve transparency in the reporting of research performed outside of researchers’ own country or community. The policy applies to researchers who have travelled to a different country to conduct research, research with Indigenous populations or their lands, and research on cultural artefacts. The questionnaire can also be requested at the journal’s discretion for any other submissions, even if these conditions are not met.  Please find more information on the policy and a link to download a blank copy of the questionnaire here: https://journals.plos.org/plosone/s/best-practices-in-research-reporting. Please upload a completed version of your questionnaire as Supporting Information when you resubmit your manuscript.

***Response: We have completed the questionnaire and it is attached. A subsection under the methods section has been added to reference the questionnaire.***

3. Thank you for stating the following in the Acknowledgments Section of your manuscript:
   We are grateful to women engaged in sex work in Southern Uganda who agreed to participate in the study; this work could not be possible without them. Special thanks to the team at the International Centre for Child Health and Development (ICHAD), who coordinated the study in Uganda with study partners, Rakai Health Sciences Program and Reach the Youth Uganda. Lastly, to the research teams at Washington University in St. Louis, Columbia University in New York, Indiana University, and New York University. Kyaterekera study is funded by the National Institute of Mental Health (NIMH) under award number R01MH116768 (MPIs: Fred Ssewamala, PhD & Susan Witte, PhD). NIMH was not involved in the study design, data collection, analysis, findings interpretation and manuscript preparation. The content in this article does not reflect the views of NIMH or the National Institutes of Health.

We note that you have provided funding information. However, funding information should not appear in the Acknowledgments section or other areas of your manuscript. We will only publish funding information present in the Funding Statement section of the online submission form. 
Please remove any funding-related text from the manuscript and let us know how you would like to update your Funding Statement. Currently, your Funding Statement reads as follows: 
Kyaterekera study is funded by the National Institute of Mental Health (NIMH) <https://www.nimh.nih.gov> under award number R01MH116768 (MPIs: FMS and SW). The funders had no role in study design, data collection and analysis, decision to publish, or preparation of the manuscript.

Please include your amended statements within your cover letter; we will change the online submission form on your behalf.

***Response: We have removed the funding statement from the manuscript. The version submitted is correct. We are not amending the statement.***

4. In your Data Availability statement, you have not specified where the minimal data set underlying the results described in your manuscript can be found. PLOS defines a study's minimal data set as the underlying data used to reach the conclusions drawn in the manuscript and any additional data required to replicate the reported study findings in their entirety. All PLOS journals require that the minimal data set be made fully available. For more information about our data policy, please see http://journals.plos.org/plosone/s/data-availability.

Upon re-submitting your revised manuscript, please upload your study’s minimal underlying data set as either Supporting Information files or to a stable, public repository and include the relevant URLs, DOIs, or accession numbers within your revised cover letter. For a list of acceptable repositories, please see http://journals.plos.org/plosone/s/data-availability#loc-recommended-repositories. Any potentially identifying patient information must be fully anonymized.

Important: If there are ethical or legal restrictions to sharing your data publicly, please explain these restrictions in detail. Please see our guidelines for more information on what we consider unacceptable restrictions to publicly sharing data: http://journals.plos.org/plosone/s/data-availability#loc-unacceptable-data-access-restrictions. Note that it is not acceptable for the authors to be the sole named individuals responsible for ensuring data access.

We will update your Data Availability statement to reflect the information you provide in your cover letter.

***Response: We acknowledge this concern. Please see below:***

***Data used in this analysis is available upon reasonable request, data access requests can be sent to any of the following Associate Deans—at Washington University’s Brown School. Provided the conditions outlined below are met, there should not be concern about data sharing. The team is open to data sharing provided the points outlined below, which were part of the study protocol, data sharing plan, and consenting process, are met.***

***Siomari Collazo-Colón, JD***

***Associate Dean for Administration***

***Hillman Hall, Room 254***

***[o] 314.935.8675 [f] 314.935.8511***

***Brown School | Washington University in St. Louis***

***[e]*** [***scollazo@wustl.edu***](mailto:scollazo@wustl.edu)

***OR***

***Fred M. Ssewamala, PhD [Also the corresponding author]***

***William E. Gordon Distinguished Professor***

***Associate Dean for Transdisciplinary Faculty Research***

***Professor of Medicine, Washington University School of Medicine***

***Goldfarb, Room 343***

***Brown School Washington University in St. Louis***

***[o] 314.935.8521 [e]*** [***fms1@wustl.edu***](mailto:fms1@wustl.edu)

- ***A formal research question is specified a priori***
- ***Names, affiliations, and roles of any other individuals who will access the shared data;***
- ***The deliverable(s)—e.g., manuscript, conference presentation—are specified a priori;***
- ***Proper credit and attribution—e.g., authorship, co-authorship, and order—for each deliverable are specified a priori.***
- ***A statement indicating an understanding that the data cannot be further shared with any additional individual(s) or parties without the PI’s permission;***
- ***IRB approval for use of the data (or documentation that IRB has determined the research is exempt)***

***The requestors are expected to handle converting electronic formats (though the research team will consider converting to tab-delimited text format if possible).***

***These conditions were prespecified in our study proposal, study protocol data sharing plan, and consenting and assenting process. Participants enrolled in the study are vulnerable women engaged in sex work, and over 40% of them living with HIV –both highly stigmatized. Thus, to protect this very vulnerable group, we stated in the consent form that only de-identified individual-level data may be shared outside of the research team and only upon completion of the conditions described above.***

5. Please review your reference list to ensure that it is complete and correct. If you have cited papers that have been retracted, please include the rationale for doing so in the manuscript text, or remove these references and replace them with relevant current references. Any changes to the reference list should be mentioned in the rebuttal letter that accompanies your revised manuscript. If you need to cite a retracted article, indicate the article’s retracted status in the References list and also include a citation and full reference for the retraction notice.

***Response: The reference list has been reviewed and it is complete and correct.***

**REVIEWERS’ COMMENTS**

**Reviewer #1: General comments:**

1. Please run the paper through a program like grammarly as there are a number of grammatical errors

***Response: The manuscript has been run through grammarly and all grammatical errors have been corrected.***

2. Write out full name of bacterial STI’s

***Response: Full names have been provided as follows: Chlamydia trachomatis, Neisseria gonorrhoeae, and Trichomonas vaginalis***

3. Why not include syphilis testing if rates in WESW are so high?

***Response: We acknowledge the reviewer’s concern. However, due to budgetary implications, we were only able to test for Gonorrhea, Trichomonas, Chlamydia and HIV. Participants who expressed concern that they might be exposed to syphilis were referred to their respective clinics or our collaborating partner facility – Rakai Health Sciences Program (RHSP) to get tested, and to receive the appropriate treatment and care, as needed.***

Intro/Methods/results/Conclusions

4. STI prevalence instead of STIs prevalence

***Response: We have addressed this throughout the manuscript.***

5. What is the general population STI prevalence?

***Response: Unfortunately, there are no national level data on the prevalence of bacterial STI in***

***the general population. Available data focus on specific subgroups, such as adolescents and***

***young women, WESW, etc. As such we are unable to provide these estimates.***

***Also see: Kakaire, O., Byamugisha, J. K., Tumwesigye, N. M., & Gamzell-Danielsson, K. (2015).***

***Prevalence and factors associated with sexually transmitted infections among HIV positive***

***women opting for intrauterine contraception. PLoS ONE, 10(4), 1–12.***

6. STI prevalence is estimated at 13% for gonorrhea, 9% chlamydia, 10% syphilis and 17% for trichomonas [4]. Is this for genital STI’s?

***Response: Yes***

7. Please give the names of the tests you used for HIV and STI

***Response: The tests have been added under “Measures: HIV and STI sample collection” as follows: Abbott Determine, Chembio statpak cassette and Abbott Bioline tests were used for HIV testing. NOVA was used to test for Neisseria gonorrhoeae, vaxpert for Chlamydia trachomatis and JD Biotech for Trichomonas vaginalis.***

8. Participants who were found to have STIs received single dose of treatment – rather say received targeted treatment for that STI.

***Response: We have changed this statement to read as suggested.***

9. Age usually reported as a median

***Response: Age was normally distributed, as such, we report the mean as it approximates the median.***

10. STI levels seem lower than expected

***Response: We acknowledge this comment.***

11. Report results consistently xx% (n= yy)

***Response: We have addressed this throughout the manuscript.***

12. Women with more than one STI were 0.7%, - this does not make sense. Rather say xx women (0.7%) tested positive for more than one bacterial STI.

***Response: We have revised this statement to read as follows: “Of the total sample, 4 (0.7%) women tested positive for more than one STI, and 32 (5.6%) tested positive for both HIV and STI.***

13. STIs prevalence is estimated at 13% for gonorrhea, 9% chlamydia, 10% syphilis and 17% for trichomonas [4]. From intro. Same sentence in discussion says syphilis is 17% with same citation.

***Response: We apologize for this oversight. We have corrected this statement to indicate the correct estimates for syphilis – at 10%.***

14. Line 294 is obscured

***Response: This was as a result of the pdf builder and has been corrected.***

15. The discussion makes some statements that seem to be more author opinion than actual study conclusions. The authors should refrain from hypothesizing reasons for associations unless these can be backed up by literature. For example saying that older women fear stigma more than young women could be completely incorrect. This is an author hypothesis and not a study result. You could say that xxx study showed this to be true and this could explain the association you found in your study. In the same way the authors should be very careful of suggesting interventions based on associations. It would be preferable to say “we noted an association between xxx and yyy and further research is needed to understand this association.

***Response: We acknowledge these concerns and have revised these statements. For the first statement, we have revised as follows:***

***“Other studies suggest that older women are often not thought about when designing HIV prevention and reduction programs, and their concerns are rarely addressed by risk reduction interventions[45] . Moreover, low education has been documented as a risk factor for sexual risk behaviours [45] and exposure to HIV and other STI among key populations, including WESW[46,47]. “***

***For the second statement, we have revised as follows:***

***“Study findings point to the need for WESW-focused efforts that address behavioural health risks to help reduce the burden of HIV and STI among this vulnerable group. Such efforts should emphasize access to user-friendly information about HIV and STIs risk reduction –taking into account their literacy levels. More economic empowerment programs addressing financial literacy gaps and access to startup capital may be important to help WESW focus on other sources of income, which may lead to better negotiating power with their customers regarding condom use.***

16. The discussion should also compare predictors of HIV in the general female population with WESW. It is understood that HIV prevalence is higher but would be interesting to understand if predictors are different.

***Response: This has been considered and we have included predictors of HIV in the general female population under the discussion section. The predictors are more less the same for the general female population and WESW.***

17. Line 298 – Prevalence of HIV tends to increase with increasing age. The trend of increasing prevalence in HIV in WESW seems to be the same as for the general population and is an epidemiological phenomenon with a disease like HIV that is incurable.

***Response: Thank you for your comment.***

18. Please provide evidence from your study supporting these statements: This may be attributed to stigma or fear of accessing preventive methods (e.g., buying condoms, PrEP and PEP services) by older women.

***Response: This statement has been revised as follows:***

***“Other studies suggest that older women are often not thought about when designing HIV prevention and reduction programs, and their concerns are rarely addressed by risk reduction interventions[45] . Moreover, low education has been documented as a risk factor for sexual risk behaviours [45] and exposure to HIV and other STI among key populations, including WESW[46,47].***

19. Line 306 – please provide evidence for the following statement: Women from households with more children may be less likely to engage in high-risk behaviours for fear that children will notice. They may also be more likely to think about the continuity of providing for their children if they are the primary caregivers, therefore taking extra precautions/preventive measures about their lives compared to those with less children

***Response: This explanation came from our community collaborative board members –including WESW. However, given the lack of documented literature, we have removed this statement from the manuscript.***

20. Line 306 should be “fewer” children. Do you have any evidence to back up your theories of why fewer children would be a risk factor for HIV. Could this be confounding?

***Response: We have removed this statement from the manuscript. Same explanation as in #19 above.***

21. Line 330 needs to be rewritten

***Response: We have revised this line to read as follows: “Women with low financial self-efficacy have less negotiating power with clients regarding safer sex, which puts them at high risk of HIV.”***

22. Line 333 – Do you mean accepting attitudes rather than approving attitudes?

***Response: Yes, this has been revised accordingly.***

23. Line 342 is incomplete

***Response: We have revised this statement to read as follows: “Such efforts should emphasize access to user-friendly information about HIV and STIs risk reduction –taking into account their literacy levels.***

24. A major limitation of this study is potential confounding. The association between low levels of education and HIV may actually be caused by poverty. Educational programs may not be helpful in this context but poverty alleviation programs may work. The authors should be careful about speculating on interventions based on associations found.

***Response: This has been considered and included in the limitations section***

**Reviewer #2:**

Although not the focus of this study, the authors did not explore the prevalence of HPV, which could have been an important opportunity for cervical cancer screening and prevention.

***Response: We acknowledge this comment, and we hope to explore HPV prevalence in the future.***

1. Were patients with a bacterial STI symptomatic?

***Response: We acknowledge this comment. However, we are unable to respond as this information was not made available to us.***

2. Were patients with a detected STI treated and with what treatment?

***Response: Yes, all participants with a detected STI were treated as follows: Azithromycin (1 gram) tablets for Neisseria gonorrhoeae and Chlamydia trachomatis, and metronidazole (2 grams) tablets for Trichomonas vaginalis. This information has been added under the “HIV and STI sample collection” section.***

3. Line 139: could the author precise the apparatus and kit use for NAAT

**Response:** ***This information has been added under the “HIV and STI sample collection” section.***

4. Line 222: The average age of participants was 31.4% in the text and 31.6% in the table 1. The status married was 25.7 in the text and 25.6 in the table 1

***Response: We apologize for this error. We have corrected this, and the text is now consistent with the figures in Table 1. Specifically, the average age of participants was 31.6. About 87% (n=473) of the participants had primary education, and only 25.6% (n=139) were married or in a relationship.***

5. Title “Sample characteristics” change for “Description and characteristics of population studied “
   - Legends of the Table: “Total Sample (N=542) % (n)” could be precise to understand that the author gives sometimes the %, or the number or the average score.

***Response: This has been revised as requested.***

6. What means the number 7.18?

***Response: This is standard deviation***
